# Changes to the cervicovaginal microbiota and cervical cytokine profile following surgery for cervical intraepithelial neoplasia

**DOI:** 10.1038/s41598-020-80176-6

**Published:** 2021-01-25

**Authors:** Rina Kawahara, Takuma Fujii, Iwao Kukimoto, Hiroyuki Nomura, Rie Kawasaki, Eiji Nishio, Ryoko Ichikawa, Tetsuya Tsukamoto, Aya Iwata

**Affiliations:** 1 Department of Obstetrics and Gynecology, Fujita Health University, School of Medicine, 1-98, Dengakugakubo, Toyoake, Aichi 470-1192 Japan; 2 Pathogen Genomics Center, National Institute of Infectious Diseases, 4-7-1, Gakuen, Musashi-murayama, Tokyo 208-0011 Japan; 3 Department of Pathology, Fujita Health University, School of Medicine, 1-98, Dengakugakubo, Toyoake, Aichi 470-1192 Japan

**Keywords:** Viral infection, Microbiome, Antimicrobial responses, Cytokines, Cervical cancer

## Abstract

Persistent HPV infection associated with immune modulation may result in high-grade squamous intraepithelial lesions (CIN)2/3. Currently, there is little information on the cervicovaginal microbiome, local cytokine levels and HPV infection related to CIN. Follow-up of patients after local surgery provides an opportunity to monitor changes in the cervicovaginal environment. Accordingly, we undertook this longitudinal retrospective study to determine associations between HPV genotypes, cervicovaginal microbiome and local cytokine profiles in 41 Japanese patients with CIN. Cervicovaginal microbiota were identified using universal 16S rRNA gene (rDNA) bacterial primers for the V3/4 region by PCR of genomic DNA, followed by MiSeq sequencing. We found that *Atopobium vaginae* was significantly decreased (*p* < 0.047), whereas *A*. *ureaplasma* (*p* < 0.022) increased after surgery. Cytokine levels in cervical mucus were measured by multiplexed bead-based immunoassays, revealing that IL-1β (*p* < 0.006), TNF-α (*p* < 0.004), MIP-1α (*p* < 0.045) and eotaxin (*p* < 0.003) were significantly decreased after surgery. Notably, the level of eotaxin decreased in parallel with HPV clearance after surgery (*p* < 0.028). Thus, local surgery affected the cervicovaginal microbiome, status of HPV infection and immune response. Changes to the cervicovaginal microbiota and cervical cytokine profile following surgery for cervical intraepithelial neoplasia may be important for understanding the pathogenesis of CIN in future.

## Introduction

Infection of the cervix by human papillomaviruses (HPVs) can cause cervical intraepithelial neoplasia (CIN). Transient HPV infections cause low-grade squamous intraepithelial lesions (CIN1), whereas persistent HPV infection presumably associated with accumulating genetic changes and immune modulation can result in high-grade squamous intraepithelial lesions (CIN2–3) or cervical cancer. Although some viral infections elicit strong host immune responses and are subsequently eliminated by the host, HPV effectively evades such immune rejection, allowing the establishment of persistent viral infection^1^.

The microbiome has a variety of functions including regulation of the immune system and metabolism of the host. The potential role of commensal cervicovaginal bacteria in modulating immune responses is largely unknown at the present time^2^. However, there is emerging evidence suggesting that the cervicovaginal microbiota is important for HPV persistence and eventually for the development of premalignant lesions^3^. Thus, we aimed to identify relationships between local host immune responses, the cervicovaginal microbiota and HPV infection in patients with CIN. However, there was a great deal of heterogeneity in the cervicovaginal microbiota among patients with CIN, possibly associated with confounding factors including smoking, number of sexual partners, sexual behavior, food intake, oral contraceptive use, and other factors. It was thus difficult to compare the relationships between HPV infections, microbiota and local host immune response in the cohort of patients as a whole, but monitoring the same individual patients with CIN over time after surgery avoid this problem of heterogeneity.

Laser cone resection, diathermy and Loop Electrosurgical Excision Procedure (LEEP) are commonly-applied local surgical interventions for CIN in developed countries. Surgery may change not only the microbial diversity^4^ but also the cervicovaginal environment, including immune responses^5^ and the status of HPV infection^6^. Thus, we undertook this retrospective longitudinal study to elucidate associations between cervicovaginal microbiota, HPV infection and cytokine profiles in individual premenopausal women with CIN before and after surgery.

## Results

### Characteristics of patients with CIN who received surgery and those under observation only

We compared the cervicovaginal microbiota in women with CIN before and after surgery to assess whether there were any correlations between bacterial composition and status of HPV infection (Figure S1). Demographic features of the enrolled patients with CIN treated by surgery or the group under observation without surgery are provided in Table 1. There were no significant differences between the two groups in multiple factors including parity and smoking. The number of different HPV genotypes was significantly decreased after surgery (*p* = 0.000), but there was no significant change over the same time period in the observation group (*p* = 0.414). In Table S1, it can be seen that 24 patients (85.7%) converted to cytology-negative (NILM) after surgery, and that 16 of 28 (57.1%) with HPV infections tested negative after surgery. The number of patients with multiple infections decreased from nine to two. In contrast, the number of patients with multiple infections did not change in the observation group.

**Table 1:** Patients’ characteristics.

Characteristics	Surgery (N = 28)				Observation only (N = 13)				*p* value
		Median	Mean	IQR		Median	Mean	IQR	
**Age (years)**	Before surgery	35.5	36.0	34–38.8	Observation 1	31	33.3	28–38	0.246^a^
	After surgery	36.5	36.9	35–39.8	Observation 2	32	34.1	28.5–38.5	0.234^a^
**Collection interval (days)**	Before surgery to After surgery	339.5	270.3	129.3–364	Observation 1 to observation 2	196	221.3	147–315	0.201^c^
**Gestation**		1	1.6	0.3–2.8		1	1.1	0–2	0.719^c^
**Parturition**		1	1.5	0–2		0	0.8	0–2	0.424^c^

		N	%			N	%		
**Smoking**	None	18	64.3			11	84.6		0.319^b^
	1–10/day	7	25			2	15.4		
	> 10/day	3	10.7			0	0		
	Brinkman index, mean (range)	62.1		0–450		18.5		0–160	0.174^c^
**HPV number (mean)**	Before surgery	1.36			Observation 1	1.69			
	After surgery	0.50			Observation 2	1.54			
	Before surgery versus after surgery	*p* value, 0.000^d^			Observation 1 versus observation 2	*p* value, 0.414^d^			

*IQR* interquartile range.

^a^Independent t-test.

^b^Pearson's chi-square test.

^c^Mann–Whitney U test.

^d^Wilcoxon signed-rank test.

### Characteristics of the cervicovaginal microbiota from patients with CIN

**Figure 1:**
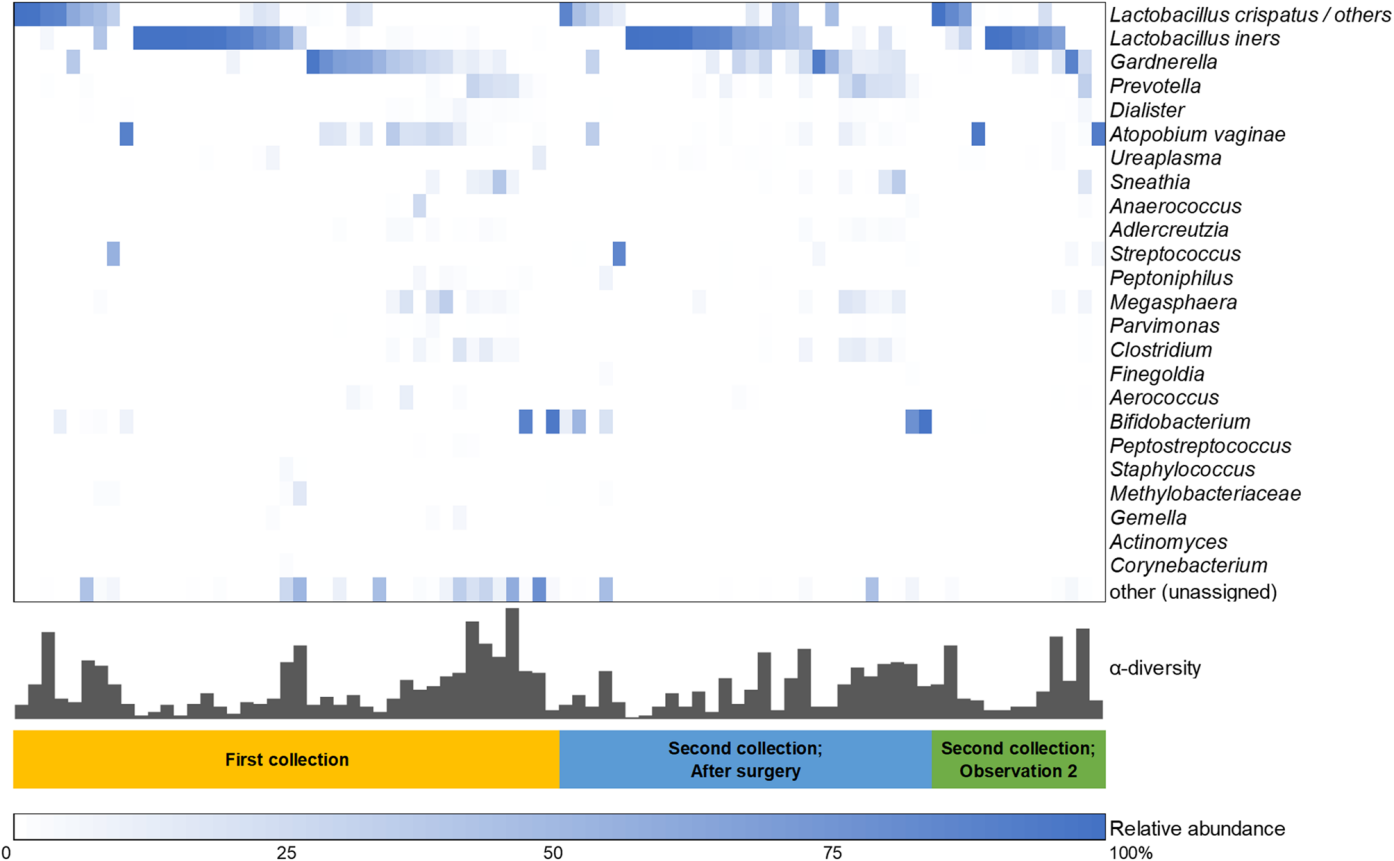
Heatmap of the relative abundance of the representative microbiota at first and second collections. Cervicovaginal microbiota were collected from 41 patients with CIN and identified by 16S rRNA V3/4 sequencing. First collection included before surgery and observation 1 described in figure. Color gradation indicates the relative abundance of microbiota. The scale of color gradation is indicated at the bottom. The number of microbial species qualified by the observed species richness (Sobs) is indicated as α-diversity in each specimen. Heatmap was drawn by Microsoft Excel.

Cervicovaginal microbiota were investigated using PCR on extracted genomic DNA with universal 16S rRNA gene (rDNA) bacterial primers for the V3/4 region followed by MiSeq sequencing. In total 2,968,275 reads were obtained from 82 specimens with an average number of reads per specimen of 36,198.5 and an average of 18.7 operational taxonomic units (OTUs) per specimen, as shown in Table 2. A total of 147 taxa was found in these specimens. At the phylum level, *Firmicutes* (65.1%), *Actinobacteria* (23.7%), *Bacteroidetes* (6.6%) and *Fusobacteria* (2.2%) were the most dominant. At the genus level, a total of 27 genera were present at an abundance > 0.1%; of these, *Lactobacillus* (56.1%), *Gardnerella* (11.3%), *Bifidobacterium* (5.9%), *Atopobium *(5.6%), and *Prevotella* (4.6%) were the dominant taxa according to the previous classification^7^. The relative abundance of the representative microbiota in the first collection and the second is depicted in Fig. 1. The most dominant species was *L. iners* in 63 specimens (76.8%). At the genus level, *Lactobacillus* and *Gardnerella* were the most abundant in both the first and second collections (Fig. 1, Table 2).

**Table 2:** Relative abundance of cervicovaginal microbiota over time.

	First collection	Second collection; After surgery	Second collection; Observation 2	Total
	N = 41	N = 28	N = 13	N = 82
**Phylum**				
Firmicutes	65.1%	64.1%	69.0%	65.5%
Actinobacteria	19.9%	27.8%	24.1%	23.7%
Bacteroidetes	8.6%	5.6%	4.2%	6.6%
Fusobacteria	2.5%	1.9%	1.9%	2.2%
**Class**				
Bacilli	55.6%	55.3%	66.3%	57.7%
Actinobacteria	15.3%	24.8%	9.6%	17.7%
Clostridia	9.4%	8.7%	2.7%	7.8%
Bacteroidia	8.6%	5.5%	4.2%	6.6%
**Order**				
Lactobacillales	55.1%	55.3%	66.1%	57.4%
Bifidobacteriales	14.2%	24.8%	9.6%	17.2%
Clostridiales	9.4%	8.7%	2.7%	7.8%
Bacteroidales	8.6%	5.5%	4.2%	6.6%
**Family**				
Lactobacillaceae	54.0%	53.4%	65.5%	56.1%
Bifidobacteriaceae	14.2%	24.8%	9.6%	17.2%
Coriobacteriaceae	4.6%	3.0%	14.5%	6.0%
Prevotellaceae	4.2%	5.5%	4.0%	4.6%
**Genus**				
Lactobacillus	46.8%	63.5%	57.5%	56.1%
Gardnerella	13.4%	10.1%	10.0%	11.3%
Bifidobacterium	5.3%	2.2%	12.7%	5.9%
Atopobium	5.9%	2.8%	9.7%	5.6%
Prevotella	4.9%	5.4%	2.9%	4.6%
**Total reads**	1,262,450	1,104,078	601,747	2,968,275
**Average number of reads/specimen**	30,791.5	39,431.4	46,288.2	36,198.5
**Average OUT/specimen**	19.3	17.3	20	18.7

*OUT* :operational taxonomic units.

### Relationships between the microbiota in the patients

**Figure 2:**
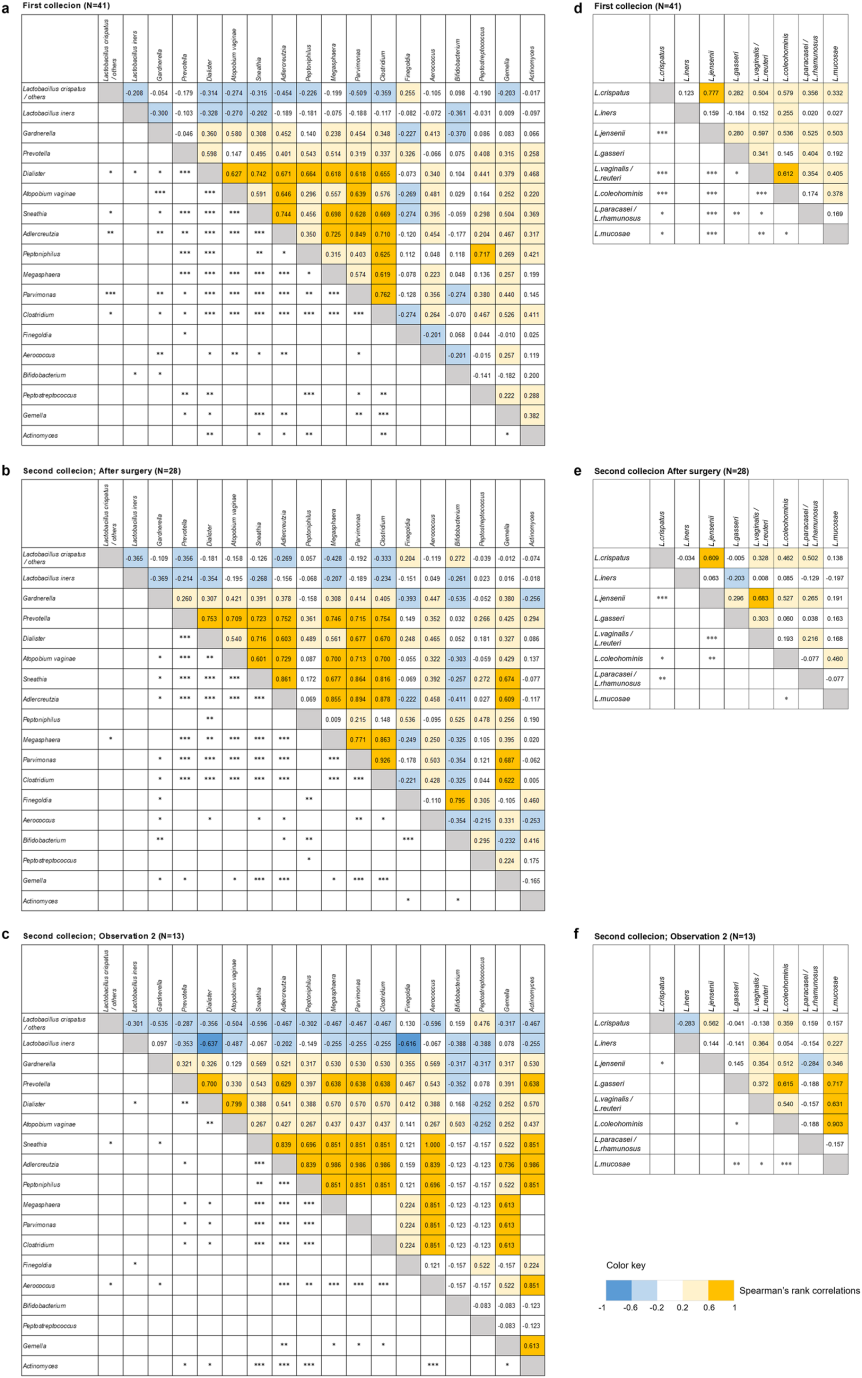
Symbiotic relationship among microbiota. Spearman’s rank correlation for multiple comparisons was estimated for each taxon as the relative abundance of each symbiont. Color and shade indicate the extent of positive and negative correlation. Dark yellow blocks indicate strong positive correlations (correlation coefficient 0.6–1.0). Pale yellow blocks indicate weak positive correlations (correlation coefficient 0.2–0.6). Pale blue indicates weak negative correlations (correlation coefficient − 0.2 to − 0.6) and dark blue strong negative correlations (correlation coefficient − 0.6 to − 1.0). Correlations were examined by QIIME2.0 (**a**–**c**). *Lactobacillus species* correlations were examined by SpeciateIT (**d**–**f**). Correlation significance: *p = 0.01–0.05, **p = 0.001–0.01, ***p < 0.001. Correlation tables were determined using Excel.

There was an inverse relationship between the presence of *L. cirspatus* and anaerobes including *Dialister, Atopobium vaginae, Adlercreutzia, Parimonas* and *Clostridium* throughout the first and second collections, as assessed by QIIME2.0 and shown in Fig. 2*.* In contrast, the presence of anaerobic bacteria including *Prevotella, Dialister, Atopobium vaginae, Sneathia, Adlercreutzia, Peptoniphilus, Megashpaera, Parvimonas* and *Clostridium* were positively correlated with each other, with no difference after surgery relative to before surgery. There was a strong correlation between *L. crispatus* and *L. jensenii* in the first collection and after surgery as determined by SpeciateIT.

### Changes in the relative abundance of microbial phyla after surgery

We compared the relative abundance of microbial phyla from patients after surgery with patients in the observation-only group. *Proteobacteria* were significantly decreased whereas *Tenericutes* were increased after surgery, as shown in Table 3 and Figure S2. There was no change over time in the observation group. At the genus level, *Atopobium vaginae* and *Methylobacteriaceae* were significantly decreased, whereas *Ureaplasma* increased after surgery.

**Table 3:** Relative abundance of microbiota; (a) phyla and (b) genus over time.

	Surgery (N = 28)			Observation only (N = 13)			Before surgery versus Observation 1 Mann–Whitney U *p* value
	Relative abundance mean % (95% CI)		Wilcoxon signed-rank test *p* value	Relative abundance mean % (95% CI)		Wilcoxon signed-rank test *p* value	
	Before surgery	After surgery		Observation 1	Observation 2		
**(a)**							
Acidobacteria	36.9 (22.9–50.9)	26.8 (15.6–37.9)	0.280	34.4 (16.9–51.9)	38.1 (12.7–63.5)	0.917	0.833
Bacteroidetes	2.5 (− 0.3–5.3)	5.6 (1.8–9.4)	0.249	4.6 (− 1.6–10.8)	0.6 (− 0.2–1.3)	0.657	0.917
Firmicutes	56.3 (42.2–70.3)	64.3 (51.9–76.7)	0.227	56 (37.1–74.9)	60.2 (34.7–85.6)	0.807	0.922
Fusobacteria	2.2 (− 0.9–5.3)	2.3 (− 0.7–5.2)	0.776	4.7 (− 2.2–11.6)	0.04 (− 0.003–0.1)	0.333	0.521
Proteobacteria	2.1 (− 1.1–5.2)	0.7 (− 0.7–2.2)	0.002*	0.2 (0.02–0.4)	0.9 (− 0.1–1.9)	0.875	0.808
Tenericutes	0.1 (− 0.03–0.2)	0.3 (0.1–0.5)	0.019*	0.001 (− 0.01–0.02)	0.1 (− 0.1–0.4)	0.144	0.506
**(b)**							
Lactobacillus crispatus/others	14.6 (4.0–25.2)	12.8 (4.1–21.4)	0.279	19.2 (− 2.7–41.1)	21.5 (− 0.8–43.7)	0.929	0.185
Lactobacillus iners	26.7 (10.2–43.2)	39.1 (23.2–55.1)	0.116	33.5 (9.2–57.9)	43 (16.9–69.1)	0.600	0.464
Gardnerella	20.1 (10.2–30.1)	14.2 (6.1–22.3)	0.247	8.6 (− 7.4–24.7)	12 (− 2.8–26.9)	0.139	0.267
Prevotella	2.0 (− 0.2–4.3)	5.5 (1.7–9.3)	0.112	4.0 (− 1.8–9.9)	2.8 (− 3.0–8.5)	0.878	0.829
Dialister	0.8 (0.2–1.4)	0.5 (0.2–0.8)	0.658	0.4 (− 0.03–0.9)	0.6 (− 0.4–1.6)	0.735	0.340
Atopobium vaginae	7.4 (3.1–11.6)	2.5 (− 0.2–5.2)	0.047*	7.4 (− 7.7–22.4)	14.9 (− 6.4–36.2)	0.237	0.405
Ureaplasma	0.1 (− 0.03–0.2)	0.3 (0.1–0.5)	0.022*	1.8 (− 0.7–4.3)	0.5 (0.05–0.9)	0.889	0.076
Sneathia	2.2 (− 0.9–5.3)	2.3 (− 0.7–5.2)	0.530	1.5 (− 0.7–3.6)	1.3 (− 1.5–4.2)	1.000	0.882
Anaerococcus	1.1 (− 0.9–3)	0.1 (− 0.1–0.3)	0.480	0.05 (− 0.01–0.1)	0.2 (− 0.2–0.6)	0.893	0.590
Adlercreutzia	0.7 (0.2–1.2)	0.6 (0.2–1.1)	0.790	0.1 (− 0.1–0.2)	0.03 (− 0.03–0.1)	0.180	0.249
Streptococcus	2.0 (− 2.1–6.1)	3.4 (− 2.9–9.7)	0.091	0.1 (− 0.01–0.1)	0.8 (− 0.4–2)	0.463	0.457
Peptoniphilus	0.5 (0.0–1.0)	0.4 (− 0.2–1)	0.347	0.1 (− 0.02–0.3)	0.05 (− 0.02–0.1)	0.080	0.872
Megasphaera	3.4 (0.2–6.5)	2.4 (0.5–4.4)	0.285	0.7 (− 0.4–1.9)	0.9 (− 0.5–2.3)	0.715	0.889
Parvimonas	0.3 (− 0.02–0.6)	0.2 (0.03–0.3)	0.878	0.1 (− 0.1–0.3)	0.1 (− 0.1–0.3)	0.655	0.413
Clostridium	1.8 (0.05–3.5)	2 (0.4–3.6)	0.799	0.7 (− 0.3–1.8)	0.1 (− 0.1–0.3)	0.180	0.393
Finegoldia	0.04 (− 0.005–0.1)	0.2 (− 0.1–0.4)	0.386	0.04 (− 0.01–0.1)	0.02 (− 0.01–0.04)	0.500	0.746
Aerococcus	0.8 (− 0.1–1.8)	0.2 (− 0.04–0.3)	0.333	0 (0.0–0.0)	0.1 (− 0.02–0.3)	0.109	0.076
Bifidobacterium	7.1 (− 2.2–16.5)	9.3 (− 0.3–19)	0.173	0.7 (− 0.8–2.2)	0.05 (− 0.1–0.2)	0.655	0.599
Peptostreptococcus	0.1 (− 0.03–0.3)	0.01 (0–0.03)	0.128	0.1 (− 0.1–0.4)	0.001 (− 0.002–0.004)	0.068	0.499
Staphylococcus	0.01 (− 0.003–0.02)	0.01 (− 0.01–0.02)	0.753	0.4 (− 0.4–1.3)	0.002 (− 0.003–0.01)	0.144	0.224
Methylobacteriaceae	0.3 (− 0.02–0.5)	0.1 (− 0.1–0.2)	0.018*	1.5 (− 1.3–4.2)	0 (0.0–0.0)	0.109	1.000
Gemella	0.3 (− 0.2–0.8)	0.02 (− 0.01–0.1)	0.345	0.2 (− 0.2–0.6)	0.01 (− 0.01–0.02)	0.655	0.712
Actinomyces	0.02 (− 0.004–0.05)	0.01 (− 0.01–0.03)	0.500	0.002 (− 0.002–0.01)	0.004 (− 0.002–0.01)	0.655	0.703
Corynebacterium	0.01 (0.0001–0.02)	0.0004 (− 0.0005–0.001)	0.075	0.2 (− 0.2–0.6)	0.01 (− 0.002–0.01)	0.715	0.466

*p < 0.05 by Wilcoxon signed-rank test.

### Correlation between microbiota and cytokines in the first collection specimen

Levels of proinflammatory cytokines including IL-1β and TNF-α were significantly increased with the presence of *anaerobics* microbiota, whereas inversely correlated with *Lactobacillus* in Table 4*.* Levels of TNF-α, IL-10 and RANTES were inversely correlated with *L. crispatus*.

**Table 4:**
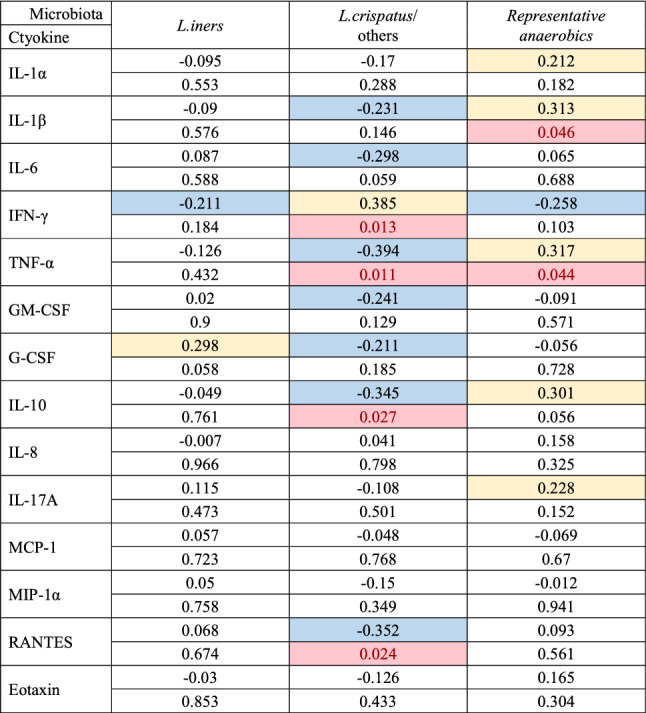
Association between microbiota and cytokines in the first collection specimens.

Upper line: correlation coefficient; lower line: significance (two-sided) *p* < 0.05 in red. Spearman’s rank correlation for multiple comparisons was calculated for the association of microbiota and levels of cytokines. Yellow and blue indicates positive and negative correlation, respectively. Pale yellow blocks indicate weak positive correlations (correlation coefficient 0.2–0.6). Pale blue blocks indicate weak negative correlations (correlation coefficient − 0.2 to − 0.6). Representative anaerobics: *Gardnerella, Prevotella, Atopobium vaginae, Sneathia, Megasphaera* and *Clostridium*.

### Changed cytokine profile after surgery

The levels of IL-1β, TNF-α, MIP-1α and eotaxin were significantly decreased after surgery but not in patients without surgery over the same time period (Table 5). Because the number of HPV genotypes detected was significantly decreased after surgery, we examined the association between the HPV infection status and cytokine profiles (Table 6). We focused on cytokine levels in patients who were positive for 7 or 13 high risk HPV genotypes before surgery but who were negative after surgery. We found that the level of eotaxin was significantly decreased in parallel with HPV negativity after surgery.

**Table 5:** Comparison of cytokine levels between groups.

Cytokines	Surgery (N = 28)						Observation only (N = 13)						Before surgery vs Observation 1 Mann–Whitney U *p* value
	No. of samples within detectable limits	Rate of specimens within detectable limits (%)	Cytokine level (pg/ml) mean	Adjustment of weighted volume (ng/mg) mean		Wilcoxon signed-rank test *p* value	No. of samples within detectable limits	Rate of specimens within detectable limits (%)	Cytokine level (pg/ml) mean	Adjustment of weighted volume (ng/mg) mean		Wilcoxon signed-rank test *p* value	
				Before surgery	After surgery					Observation 1	Observation 2		
IL-1α	56/56	100.0	814.3	5,566.0	5,513.8	0.767	26/26	100.0	873.4	8,454.1	3,808.2	0.075	0.911
IL-1β	56/56	100.0	1,983.6	17,749.1	10,901.6	0.006*	26/26	100.0	1,252.7	12,683.2	7,458.4	0.917	0.069
IL-6	56/56	100.0	540.9	4,482.2	3,012.5	0.056	26/26	100.0	653.4	4,664.1	5,997.2	0.345	0.519
IFN-γ	13/56	23.2	1.1	6.4	12.6	0.155	9/26	34.6	13.4	167.7	29.8	0.575	0.094
TNF-α	43/56	76.8	47.8	461.1	182.2	0.004*	21/26	80.8	26.2	296.4	174.2	0.814	0.062
GM-CSF	4/56	7.1	0.1	0.7	0	0.068	1/26	3.8	0.0	0.1	0	0.317	0.522
G-CSF	56/56	100.0	506.2	3,466.0	3,418.7	0.964	26/26	100.0	487.1	3,667.5	3,386.0	0.917	0.845
IL-10	42/56	75.0	14.9	84.5	106.3	0.073	21/26	80.8	10.9	123.7	70.1	0.657	0.197
IL-8	56/56	100.0	28,852.9	222,134.0	215,198.7	0.053	26/26	100.0	30,307.9	253,656.5	225,098.3	0.917	0.466
IL-17A	6/56	10.7	0.7	6.4	1.1	0.463	2/26	7.7	0.2	6.0	0	0.180	0.927
MCP-1	56/56	100.0	520.7	3,402.7	3,429.1	0.452	26/26	100.0	660.5	3,451.8	5,020.0	0.173	0.466
MIP-1α	56/56	100.0	122.1	955.5	856.0	0.045*	26/26	100.0	213.7	2,977.3	800.9	0.701	0.634
RANTES	53/56	94.6	73.7	434.1	679.8	0.946	26/26	100.0	77.8	1,515.8	128.1	0.382	0.556
Eotaxin	12/56	21.4	1.2	14.5	0.1	0.003*	3/26	11.5	0.3	4.2	0	0.109	0.274

Cytokine levels assessed according to our report^25^ and adjusted by weighted volume in the cervical mucus.

*p < 0.05 by Wilcoxon signed-rank test.

**Table 6:** Time course for cytokine levels in patients with 7 or 13 high-risk HPV genotypes before surgery, but none after surgery.

Cytokines	Seven high-risk HPV genotypes in Japan (N = 13)						Thirteen high-risk HPV genotypes worldwide (N = 16)					
	No. of samples within detectable limits	Rate of specimens within detectable limits (%)	Cytokine level (pg/ml) mean	Adjustment of weighted volume (ng/mg) mean		Wilcoxon signed-rank test *p* value	No. of samples within detectable limits	Rate of specimens within detectable limits (%)	Cytokine level (pg/ml) mean	Adjustment of weighted volume (ng/mg) mean		Wilcoxon signed-rank test *p* value
				Before surgery	After surgery					Before surgery	After surgery	
IL-1α	26/26	100.0	1014.4	6,676.8	7,086.9	0.382	32/32	100.0	956.9	6,329.7	6,906.2	0.255
IL-1β	26/26	100.0	2,485.3	19,260.6	17,985.8	0.600	32/32	100.0	2,154.4	16,937.5	15,441.4	0.362
IL-6	26/26	100.0	590.5	3,994.8	3,802.7	0.917	32/32	100.0	550.6	3,996.4	3,426.3	0.501
IFN-γ	7/26	26.9	0.9	0.7	18.7	0.463	8/32	25.0	1.3	10.9	15.2	0.866
TNF-α	20/26	76.9	50.7	344.2	328.1	0.286	25/32	78.1	46.1	335.9	283.0	0.124
GM-CSF	2/26	7.7	0.1	0.9	0	0.180	3/32	9.4	0.1	1.1	0	0.109
G-CSF	26/26	100.0	448.6	2,738.0	3,127.4	0.552	32/32	100.0	467.2	3,099.2	3,253.6	0.836
IL-10	20/26	76.9	20.3	61.8	173.0	0.929	24/32	75.0	18.5	75.2	148.8	0.551
IL-8	26/26	100.0	34,495.0	240,894.6	309,049.1	0.552	32/32	100.0	32,249.8	227,265.3	289,580.8	0.679
IL-17A	3/26	11.5	1.3	13.0	0	0.109	3/32	9.4	1.0	10.6	0	0.109
MCP-1	26/26	100.0	369.7	2,278.0	2,564.7	0.463	32/32	100.0	422.9	3,104.6	2,517.1	0.179
MIP-1α	26/26	100.0	148.9	801.9	1,413.0	0.600	32/32	100.0	144.0	907.5	1,288.2	0.959
RANTES	24/26	92.3	45.6	253.7	398.6	0.279	29/32	90.6	76.9	296.4	863.6	0.326
Eotaxin	7/26	26.9	1.6	19.4	0.2	0.028*	7/32	21.9	1.3	15.8	0.2	0.028*

Seven high-risk HPV genotypes in Japan; HPV 16, 18, 31, 33, 45, 52, and 58. 13 high-risk HPV genotypes worldwide; HPV 16, 18, 31, 33, 35, 39, 45, 51, 52, 56, 58, 59, and 68. *p < 0.05 by Wilcoxon signed-rank test.

## Discussion

Local interplay between the microbiome and the immune response may be important for understanding the pathogenesis of the sequence of events from HPV infection, CIN development and progression to cervical cancer^8^. As one approach to investigating this issue, we examined how removal of the neoplastic lesions by surgery impacted on associations between microbiome diversity, local immune responses and the number of HPV genotypes in patients with CIN. This showed that *Atopobium* was significantly decreased after surgery (Table 3 and Figure S2), in parallel with the decreased number of HPV genotypes detected after surgery. *Atopobium* was positively correlated with the presence of anaerobic bacteria (Fig. 2) and with HPV persistence^9^. Both *Atopobium* and *Gardnerella* were associated with CIN^10^*.* Decreased HPV infections and removal of neoplastic lesions might be associated with decreased diversity of microbiota^10–13^. Thus, the presence of CIN lesions could contribute to the maintenance of microbiome diversity^3^.

*Ureaplasma* was found in the vagina or cervix of 40–80% of premenopausal asymptomatic women^14^ but were also reported to be associated with CIN^15^. Our data showed that *Ureaplasma* increased after surgery. One explanation for this could be that surgical intervention increases the opportunity for *Ureaplasma* growth or that the environment before surgery was not appropriate for this species due to competition from other microbes. *Prevotella* may provide nutrients such as ammonia and amino acids to other members of the microbial community such as *Gardnerella* and *Peptostreptococcus*^16^ and assume a role as the hub for vaginal microbiota^17^. Indeed, there was a positive correlation between the presence of *Prevotella* and other anaerobics regardless of surgery. *Prevotella* could therefore be critical for maintenance of a dysbiome in the vagina (Fig. 2).

Using QIIME2.0, we separated *Lactobacillus* into *L. crispatus, L. iners* and unclassified *L. spp* and employed SpeciateIT for species of *Lactobacillus* classification. *L. crispatus* is the most common vaginal H_2_O_2_-producing * Lactobacillus* species, followed by *L. jensenii*, whereas *L. iners* does not produce H_2_O_2_ (which has been associated with increased risk of abnormal vaginal microbiota)^18^. In vitro experiments demonstrated that *L. iners* and *Gardnerella* disrupt the cervical epithelial barrier by regulating adherens junction proteins, cervical immune responses and miRNA expression, whereas *L. crispatus* has a protective effect^19^. In our analysis, the presence of *L. crispatus* was positively correlated with *L. jensenii,* and *L. crispatus* and others including *L. jensenii* were negatively correlated with anaerobics (Fig. 2).

High levels of proinflammatory cytokines were associated with the presence of anaerobic bacteria and inversely correlated with the presence of *Lactobacillus* (Table 4). High levels of multiple proinflammatory cytokines were strongly associated with highly diverse bacterial communities in patients, suggesting that specific genital bacteria induced a robust local immune response^2^. *Atopobium* was reported to induce strong expression of IL-1β and TNF-α in cultured cells^20^. In other studies, high levels of IL-1β were associated with bacterial vaginosis^21^ or cervical dysplasia^22^. In contrast, TNF-α, IL-10, and RANTES were down-regulated in bacterial vaginosis in patients where *L. crispatus* dominated. The level of TNF-α was decreased post-LEEP compared with patients without LEEP^5^. In addition to TNF-α, we found that IL-1β, MIP-1α and eotaxin were decreased after surgery (Table 5). MIP-1α was reported to be a biomarker for precursor lesions in cervical cancer^22–24^. HPV clearance after surgery was inversely correlated with the level of eotaxin. This is consistent with a report that eotaxin was measurable in cultured cervical cells with integrated HPV16/18 genomes^25^. Marks observed high levels of eotaxin in the cervical mucus of patients with HPV infections^26^. One possible explanation for this finding is that precursor lesions infected with HPVs produce eotaxin. High expression of IFN-γ was correlated with *L. crispatus* for unknown reasons. However, the cause of the high expression of proinflammatory cytokines remains controversial because cervical neoplasia, bacterial vaginosis and HPV infections are known to be factors influencing each other.

The present study was rigorous in the diagnosis, recruitment, treatment and management of patients, and specimens were all taken by a single colposcopist. Clinical data including histology, cytology and HPV genotypes were precisely recorded (Table S1). Yin reported on the effects of surgery on TNF-α expression, as a biomarker of inflammation^27^. The level of TNF-α was increased one month after surgery, and decreased again thereafter. It takes approximately 6 months to heal the wound of the LEEP surgery^5^. The patients received limited oral antibiotics and anti-inflammatory drugs for two days after surgery. To avoid interference caused by the surgery per se, specimens were taken at a mean of 280 days, and the interval of IQR was 129–364 days, as shown in Table 1. Therefore, we believe that inflammation or drugs did not affect the microbial environments and cytokine profiles. The rate of residual HPV DNA 7–9 months after conization was < 10% as reported by Kim et al^6^. Therefore, specimens were taken 7–9 months after surgery in this aspect. Of note, some patients remained HPV-positive whatever neoplastic lesion was removed (Table S1). It is therefore necessary to be aware of the issue of late recurrence. The etiology of multiple infection in terms of cervical carcinogenesis is unknown. Multiple infections result from many different factors including an immunocompromised state or increased chances of infection from multiple sex partners. Multiple infections are often observed in LSIL, but thereafter monoclonal cells infected with a certain high-risk HPV genotype grow rapidly, whereas cells infected with other HPV genotypes decrease, possibly as a result of immune responses. Consequently, infection with a single strain is usually observed in HSIL or cancer. This is likely the reason why the number of different HPV genotypes was decreased or reduced to zero by surgery. The elimination or diminished HPV genotypes after surgery is possibly due to the resection of the infected lesion. However, HPV infections might be present beyond the surgical area. Whether or not these are eliminated would depend on the individual immune response.

Pre- or post-menopausal status is a critical factor influencing diversity of the cervicomicrobiome^28^. Young healthy women had dominant *L. crispatus* or *L. iners* communities^29^, whereas postmenopausal women had a paucity of *Lactobacillus* and dominant *Streptococcus*, *Prevotella*^17^ and *Atopobium*^28^. Cervical mucus is more abundant in young women; the frequency of CIN peaks in women in their 30’s. LEEP, diathermy and laser cone treatments are the most appropriate options for fertility-sparing surgery in women of childbearing age. Taking these factors together, we enrolled premenopausal women, mainly in their 30′s in order to exclude an age-associated effect on the reduction of cervical mucus. Ravel reported that there are differences in the vaginal microbiome according to race^29^. Human immunodeficiency virus-infected individuals represent a unique cohort of patients with HPV infections at increased risk of developing cervical cancer. We therefore recruited immunocompetent Japanese patients for the present study. We also fixed the anatomical site of the sampling lesion and the sampling devices because of differences resulting from using different methods^30,31^.

There are some limitations to this study. We showed sequencing results in Table 2, but these could be different if the target region of 16s rRNA genes analyzed or methods themselves were different^32^. There may also be some selection bias for the enrolled patients. Patients with CIN2 and tiny lesions were apt to be assigned to observation only, whereas patients with CIN3 and larger lesions were assigned to surgery. However, there was in fact little difference between them, because the HSIL category in pathology includes both CIN2 and CIN3. Another limitation is the lack of adjustment for risk factors and possible confounders between groups. Tobacco smoking is a risk factor for bacterial vaginosis, and *Peptostreptococcus* and *Veillonella* are associated with smoking^33,34^. Smoking is associated with a lower proportion of *Lactobacillus* than observed in non-smokers^35^. However, there was no difference between the surgery and observation groups regarding smoking (Table 1). The effect of smoking was not seen here, possibly due to the small number of patients. A further limitation is that the time course of sample taking was limited. Brotman reported an average of 29 samples per participant to examine the association between HPV infections and the vaginal microbiome^12^. Fluctuations over time of the cervicovaginal microbiota from the same individual were observed^36^. We did not determine the status of bacterial vaginosis including pH and Nugent Score. We have no patients with recurrence nor progression of CIN. Therefore, we have not examined the association between CIN pathogenesis and vaginal environment possibly due to the short observation period. A larger cohort and multiple longitudinal clinical studies will be needed to expand and validate our findings. Moreover, mechanistic studies using in vitro and in vivo models will be required to fully understand the complex relationships among the cervicomicrobiome, HPV infections, local immune responses and regression-vs-persistence-vs-progression of neoplastic disease of the cervix.

In summary, we have undertaken a longitudinal retrospective study to determine associations between the cervicovaginal microbiota, HPV infections and cytokine profiles in premenopausal patients with CIN, comparing patients receiving surgery with those under observation only. The dominant microbiota in Japanese premenopausal patients with CIN was *L. iners*, the abundance of which was unchanged by surgery. There was an inverse relationship between *L. crispatus* and the presence of anaerobic bacteria. At the genus level, *Atopobium vaginae* was significantly decreased, whereas *Ureaplasma* increased after surgery. We found that high levels of proinflammatory cytokines including IL-1β and TNF-α were significantly increased in parallel with the presence of anaerobics, and inversely correlated with *Lactobacillus* dominance. Levels of IL-1β, TNF-α, MIP-1α and eotaxin were significantly decreased after surgery. Of note, the expression of eotaxin in parallel with HPV clearance after surgery was significantly decreased. In conclusion, we found that surgical intervention dramatically changed the cervicovaginal microbiome and local immune responses. We could find the association among microbiota, HPV and local immune response with the recurrence or progression of CIN in future.

## Materials and methods

### Study subjects

Specimens were collected from 41 Japanese patients with CIN, aged 24–48 years (median 35), who attended the outpatient clinic at Fujita Health University Hospital, Aichi Prefecture, Japan, for routine gynecological examinations from April 2016 to March 2019. Histology results were classified according to the Richart classification. Cytological interpretation was classified according to the Bethesda 2001 system.

The patients were divided into a group requiring surgery (n = 28) and observation only (n = 13) according to the clinical decision-making process (Figure S1). Of the former, five underwent laser cone resection and 23 LEEP with diathermy. First collection of specimens designated “before surgery” or “observation 1” was from all patients. The second collection designated “after surgery” or “observation 2” was taken from each group. At first collection, disease as classified by histology into chronic cervicitis (n = 1), five CIN1, 23 CIN2 and 12 CIN3. Cytology results were classified as negative for intraepithelial lesions or malignancy (NILM) (n = 1), five atypical squamous cells of undetermined significance (ASC-US), eight low-grade squamous intraepithelial lesions (LSIL), 24 high-grade squamous intraepithelial lesions (HSIL), and three atypical squamous cells, cannot exclude high-grade squamous intraepithelial lesions (ASC-H). In the “after surgery” group, there were 24 NILM, one ASC-US, one LSIL, and two HSIL. In the “observation 2” group, histological classification was chronic cervicitis (n = 1), three CIN1, three CIN2, five CIN3 and one not determined. Cytology results were one NILM, one LSIL, and two HSIL.

We excluded patients who (a) were younger than 20 years or older than 49 years; (b) were pregnant or postmenopausal; (c) had undergone previous treatment with chemotherapy, radiation, or surgery for CIN; (d) had cancer; or (e) took medication for sexually transmitted diseases. Specimens taken during menstrual period were excluded. The study protocol was approved by the Ethics Committees of Fujita Health University and the National Institute of Infectious Diseases. Written informed consent was obtained from each patient. All the methods were performed in accordance with the relevant guidelines and regulations. The anatomy site of the sample taking was fixed. Cervical mucus specimens were collected using BD BBL Culture Swab (Becton, Dickinson and Company, Franklin Lakes, NJ, USA) for microbial analysis and using Merocel cervical sponges (Medtronic Xomed, Inc., Jacksonville, FL, USA) for cytokine analysis and stored at − 80 °C. The cervical brush was inserted into the cervical canal to collect ectocervical and endocervical cells for HPV genotypes and stored at − 80 °C.

### HPV genotyping

For HPV genotyping assays, total DNA was extracted with the QIAamp DNA Mini Kit (QIAGEN GmbH, Hilden, Germany). Each cytobrush was soaked in 400 μl of phosphate-buffered saline, according to the manufacturer’s protocol for swabs. The extracted DNA was eluted with 120 μl Buffer AE. Quality of extracted DNA was determined spectrophotometrically using the NanoDrop ND-1000 (NanoDrop Technologies Inc., Wilmington, USA). HPV genotyping assays were performed by polymerase chain reaction (PCR) with PGMY primers followed by reverse line blot hybridization^37^. This assay can detect the 31 HPV genotypes, HPV 6, 11, 16, 18, 26, 31, 33, 34, 35, 39, 40, 42, 44, 45, 51, 52, 53, 54, 55, 56, 57, 58, 59, 66, 68, 69, 70, 73, 82, 83 and 84. Details of the pathological results and HPV genotypes are shown in Table S1. Two groups of HPV genotypes were selected for further analysis: seven most common high-risk HPV genotypes in Japan (HPV 16, 18, 31, 33, 45, 52, and 58), and 13 genotypes (HPV 16, 18, 31, 33, 35, 39, 45, 51, 52, 56, 58, 59, and 68) found worldwide.

### DNA extraction for microbial analysis

DNA was extracted from cervicovaginal mucus, collected using a BD BBL Culture Swab Plus with a ChargeSwitch Forensic DNA Purification Kit (Thermo Fisher Scientific, Waltham, MA, USA) following the manufacturer’s instructions. DNA concentrations were measured with a Synergy H1 microplate reader (BioTek, Winooski, VT) and a QuantiFluor dsDNA system (Promega, Madison, WI, USA) following the manufacturer’s instructions.

### Library preparation and sequencing

Cervicovaginal microbiota were determined using extracted genomic DNA by PCR with universal 16S rRNA gene (rDNA) bacterial primers for the V3/4 region followed by MiSeq sequencing. Libraries were prepared by a two-step tailed PCR method. First, two PCR analyses were conducted, the first with Bakt_341F and Bakt_805R primers^38^, the second with index primers. Library concentrations were measured using a Synergy H1 microplate reader (BioTek) and a QuantiFluor dsDNA System (Promega), and library quality was assessed with a Fragment Analyzer (Advanced Analytical Technologies, Ankeny, IA, USA) and a dsDNA 915 Reagent Kit (Agilent, Santa Clara, CA, USA), following the manufacturer’s instructions. Paired-end sequencing (2 × 300 bp) was carried out on the Illumina MiSeq platform (Illumina, San Diego, CA, USA) with the MiSeq Reagent Kit v3 (Illumina).

### Microbial data analysis

Reads that began with a sequence that completely matched the primer used were extracted by using the fastx_barcode_splitter tool in the FASTX-Toolkit, and then the primer sequence was trimmed. Next, the Sickle tool^39^ with a quality value of 20 was used to trim and filter the reads; trimmed reads and paired-end reads with fewer than 150 bases were discarded. The FLASH paired-end merge script^40^ was used to merge the remaining reads under the following conditions: fragment length after merge, 420 bases; read fragment length, 280 bases; and minimum overlap length, 10 bases. All merged sequences were used for further analysis.

QIIME2.0 (2019.4), with the default parameter values, was used for sequence denoising using the DADA2 method, for chimera checking and then for taxonomic assignments with the Greengenes database (13_8) clustered at 97% identity^41,42^.

RDP classifier was used for taxonomic assignments for the genus Lactobacillus; the merged sequences (reads) were used as the input to the RDP classifier. Because the region of the gene to be analyzed was different and a new database had to be created, the 16S rRNA gene sequences of 12 species of the genus *Lactobacillus* (*L. coleohominis, L. crispatus*, *L. gasseri, L. iners, L. jensenii*, *L. mucosae*, *L. paracasei*, *L. paraplantarum*, *L. plantarum*, *L. reuteri*, *L. rhamnosus*, and *L. vaginalis*) included in the database attached to SpeciateIT (https://sourceforge.net/projects/speciateit/) were downloaded from the Ribosomal Database Project website (http://rdp.cme.msu.edu/hierarchy/hb_intro.jsp) using the following options: strain = both; source = isolates, size ≥ 1,200 bases; quality = good, and taxonomy = nomenclatural. Following SpeciateIT instructions, a database for species discrimination analysis was created from the 16S rRNA gene sequences, and then a species discrimination analysis was performed using SpeciateIT, with the created database and the output sequences of RDP classifier classified as the genus Lactobacillus. Alpha diversity estimators observed species richness (Sobs)—the observed OTUs was calculated for the overall bacterial community using QIIME2.0.

### Protein extraction from cervical sponges

Protein for cytokine analysis was extracted from Merocel cervical sponges, using previously described methods^25^. First, the wet weight of each sponge was recorded, and each was then placed in a 2-ml Spin-X centrifuge filter tube (Corning Inc., Corning, NY, USA), and 300 μl of extraction buffer containing PBS(Sigma-Aldrich, St. Louis, MO, USA), with the addition of 256 mM NaCl and 100 μg/ml aprotinin (Wako, Amagasaki, Japan) were slowly added. The sponges were incubated at 4 °C for 2 h and then centrifuged at 14,000 rpm for 15 min at 4 °C, followed by the addition of 30 µl of fetal bovine serum to the 270 µl of extract. The sample was then vortexed briefly, aliquoted, and frozen at − 80 °C until further testing. The remaining extracts were stored at − 80 °C until the time of total protein measurement.

### Cytokine measurements using cytometric bead array

Measurements of cytokine levels were as reported previously^25^. The following cytokines, chemokines, and growth factors were measured using multiplexed bead-based immunoassays (Cytometric Bead Array; CBA) according to the manufacturer’s protocol (BD Biosciences): interleukin (IL)-1α (Cat# 560153), IL-1β (Cat# 558279), IL-6 (Cat# 558276), IFN-γ (Cat# 558269), tumor necrosis factor (TNF)-α (Cat# 558273), granulocyte–macrophage colony-stimulating factor (GM-CSF) (Cat# 558335), granulocyte colony-stimulating factor (G-CSF) (Cat# 558326), IL-10 (Cat# 558274), IL-8 (Cat# 558277), IL-17A (Cat# 560383), monocyte chemoattractant protein (MCP)-1 (Cat# 558287), macrophage inflammatory protein (MIP)-1α (Cat# 558325), RANTES (Cat# 558324), and eotaxin (Cat# 558329). Cervical extracts were thawed and diluted 1:1 to 1:1000 in extraction buffer depending on the cytokine levels. Briefly, a 10-point standard curve ranging from 0 to 2500 pg/ml for each cytokine was prepared using the cytokine standard provided in each kit. Samples and cytokine standards were incubated in the capture bead mixture for 1 h and phycoerythrin-conjugated antibodies against each cytokine were added to the sample-bead mixture for 2 h of incubation at room temperature. All buffers used were from the CBA human soluble protein master buffer kit (Cat# 558265, BD Biosciences). Beads were washed and analyzed using a BD FACSCalibur flow cytometer (BD Biosciences). Mean fluorescence intensity for each bead cluster was converted into cytokine concentrations based on the 10-point standard curve, using FCAP Array software (BD version 3.0.1).

### Adjustment of cytokine levels

Cytokine levels were adjusted by weighted volume, according to a previous report^25,43^. To compare differences in sponge weights after specimen collection, the dilution factor was calculated as [(x − y) + 300 mg of buffer]/(x − y), where x equals the weight of the sponge after collection and y is the weight of the dry sponge. Each cytokine measured was multiplied by this dilution factor to obtain weight-normalized values.

### Statistical analysis

All statistical analyses were performed using SPSS for Windows (ver. 22.0.0.0; IBM Corp, Armonk, NY, USA). Mann–Whitney U tests after Bonferroni correction were used to compare continuous data between groups. To compare before with after intervention, the Wilcoxon signed-rank test was used. Spearman’s rank correlation for multiple comparisons was estimated for 1) the presence of each taxon and 2) the association between cytokine levels and microbiota. We defined *p* < 0.05 as significant.

## Supplementary information

Supplementary Caption.

Supplementary Table.

Supplementary Figure 1.

Supplementary Figure 2.
