# Supplementary material for: Changes to the cervicovaginal microbiota and cervical cytokine profile following surgery for cervical intraepithelial neoplasia
Source: Sci Rep. 2021 Jan 25;11:2156. doi: 10.1038/s41598-020-80176-6 (PMC7835242; doi:10.1038/s41598-020-80176-6)
Supplement: Supplementary file 1 — Supplementary Caption. [file 41598_2020_80176_MOESM1_ESM.docx]

Changes to the cervicovaginal microbiota and cervical cytokine profile following surgery for cervical intraepithelial neoplasia

Rina Kawahara^1,¶^,Takuma Fujii^1,^*^,¶^, Iwao Kukimoto^2^, Hiroyuki Nomura^1^, Rie Kawasaki^1^, Eiji Nishio^1^, Ryoko Ichikawa^1^, Tetsuya Tsukamoto^3^, Aya Iwata^1^

^1^Department of Obstetrics and Gynecology, ^3^Department of Pathology, Fujita Health University, School of Medicine, 1-98, Dengakugakubo, Toyoake, Aichi, 470-1192, Japan

^2^Pathogen Genomics Center, National Institute of Infectious Diseases, 4-7-1, Gakuen, Musashi-murayama, Tokyo, 208-0011, Japan

*corresponding author

E-mail: [fujiit44@fujita-hu.ac.jp](mailto:fujiit44@fujita-hu.ac.jp) (FT)

**ORCID ID** (Takuma Fujii): 0000-0002-7233-8663

^¶^These authors contributed equally to this work.

**Supplementary data:**

**Figure S1**

**Timing of the collection of the specimens**

Patients with CIN were divided into those for surgery or observation only. Specimens were collected twice from each patient.

**Figure S2**

**Association between relative abundance of microbiota and timing of the collection of the specimen; phyla (a) and genus (b).**

The y-axis indicates the relative abundance. The x-axis indicates timing of specimen collection.

*indicates statistically significant:*p*<0.05

**Table S1**

**Histology and cytology results, and HPV genotyping, of the first and second collection specimens.**

Supplementary information: two supplementary figures and one table are included in this article.
