# Supplementary material for: Changes to the cervicovaginal microbiota and cervical cytokine profile following surgery for cervical intraepithelial neoplasia
Source: Sci Rep. 2021 Jan 25;11:2156. doi: 10.1038/s41598-020-80176-6 (PMC7835242; doi:10.1038/s41598-020-80176-6)
Supplement: Supplementary file 2 — Supplementary Table. [file 41598_2020_80176_MOESM2_ESM.docx]

| Table S1. Patient list of the histology, cytology results and status of HPV infections in the first and second collections | | | | | | | | | | | | |
| --- | --- | --- | --- | --- | --- | --- | --- | --- | --- | --- | --- | --- |
| First collection (N=41) | | | | | Second collection (N=41) | | | | | Group | Surgery day to Second collection interval, days | Observation first collection to Second collection interval, days |
|  |  |  |  |  |  |  |  |  |  |  |  |  |
| ID | Age | Histology | Cytology | HPV | ID | Age | Histology | Cytology | HPV |  |  |  |
|  |  |  |  |  |  |  |  |  |  |  |  |  |
| Sc-0569 | 34 | CIN3 | HSIL | 51 | Sc-0732 | 35 | - | NILM | negative | Surgery | 364 | - |
| Sc-0505 | 31 | CIN3 | HSIL | 16 | Sc-0561 | 32 | - | HSIL | negative | Surgery | 107 | - |
| Sc-0634 | 33 | CIN2 | ASCUS | 16 | Sc-0700 | 33 | - | NILM | negative | Surgery | 182 | - |
| Sc-0470 | 36 | CIN2 | HSIL | 16 | Sc-0559 | 37 | - | NILM | negative | Surgery | 91 | - |
| Sc-0516 | 30 | CIN3 | LSIL | 56, 82 | Sc-0644 | 30 | - | NILM | 56, 82 | Surgery | 203 | - |
| Sc-0638 | 34 | CIN3 | HSIL | 16 | Sc-0726 | 35 | - | NILM | negative | Surgery | 142 | - |
| Sc-0620 | 36 | CIN2 | HSIL | 51, 68 | Sc-0770 | 37 | - | NILM | 51 | Surgery | 364 | - |
| Sc-0504 | 29 | CIN2 | HSIL | 52, 58 | Sc-0639 | 30 | - | NILM | negative | Surgery | 406 | - |
| Sc-0503 | 36 | CIN3 | HSIL | 52 | Sc-0667 | 37 | - | NILM | 52 | Surgery | 420 | - |
| Sc-0557 | 41 | CIN2 | ASU-US | 16, 53, 69 | Sc-0698 | 43 | - | NILM | negative | Surgery | 343 | - |
| Sc-0595 | 39 | CIN2 | HSIL | 31, 58 | Sc-0710 | 40 | - | HSIL | 58 | Surgery | 224 | - |
| Sc-0538 | 38 | CIN1 | LSIL | 16 | Sc-0585 | 38 | - | NILM | negative | Surgery | 98 | - |
| Sc-0410 | 46 | CIN3 | NILM | negative | Sc-0586 | 48 | - | LSIL | 45 | Surgery | 121 | - |
| Sc-0461 | 35 | CIN1 | LSIL | 33, 52 | Sc-0601 | 36 | - | NILM | negative | Surgery | 364 | - |
| Sc-0479 | 43 | CIN3 | HSIL | 66 | Sc-0618 | 44 | - | NILM | HPVX | Surgery | 364 | - |
| Sc-0554 | 31 | CIN2 | HSIL | 16, 39, 59 | Sc-0627 | 31 | - | NILM | 39 | Surgery | 112 | - |
| Sc-0495 | 37 | CIN3 | ASC-H | 51 | Sc-0631 | 38 | - | NILM | negative | Surgery | 364 | - |
| Sc-0522 | 34 | CIN2 | HSIL | 16, 52 | Sc-0641 | 35 | - | NILM | 81 | Surgery | 350 | - |
| Sc-0580 | 35 | CIN3 | ASC-H | 31 | Sc-0645 | 36 | - | NILM | 18 | Surgery | 125 | - |
| Sc-0533 | 38 | CIN2 | HSIL | 51 | Sc-0655 | 39 | - | ASC-US | 42, 51 | Surgery | 168 | - |
| Sc-0543 | 34 | CIN3 | HSIL | 16 | Sc-0659 | 35 | - | NILM | negative | Surgery | 289 | - |
| Sc-0643 | 35 | CIN3 | ASC-H | 16 | Sc-0691 | 35 | - | NILM | 16 | Surgery | 91 | - |
| Sc-0568 | 42 | CIN2 | HSIL | 16 | Sc-0718 | 43 | - | NILM | negative | Surgery | 364 | - |
| Sc-0547 | 33 | CIN2 | ASU-US | 16, 44 | Sc-0724 | 34 | - | NILM | negative | Surgery | 420 | - |
| Sc-0555 | 34 | CIN2 | HSIL | 16 | Sc-0734 | 35 | - | NILM | 16 | Surgery | 427 | - |
| Sc-0612 | 36 | CIN2 | LSIL | 58 | Sc-0755 | 37 | - | NILM | negative | Surgery | 364 | - |
| Sc-0622 | 39 | CIN2 | HSIL | 31 | Sc-0768 | 40 | - | NILM | negative | Surgery | 336 | - |
| Sc-0624 | 40 | CIN2 | HSIL | 35 | Sc-0789 | 41 | - | NILM | negative | Surgery | 364 | - |
| Sc-0528 | 48 | CIN2 | HSIL | 52 | Sc-0579 | 49 | CIN3 | HSIL | 52 | Observation | - | 140 |
| Sc-0576 | 30 | CIN2 | HSIL | 6, 16, 33, 51, 58 | Sc-0694 | 31 | CIN3 | - | 6, 33, 51, 58 | Observation | - | 322 |
| Sc-0704 | 38 | CIN2 | HSIL | 16 | Sc-0776 | 38 | CIN3 | - | 16 | Observation | - | 147 |
| Sc-0715 | 32 | CIN2 | HSIL | 52 | Sc-0780 | 33 | CIN3 | - | 52 | Observation | - | 147 |
| Sc-0615 | 28 | CIN2 | LSIL | 58, 68 | Sc-0677 | 29 | CIN2 | LSIL | 68 | Observation | - | 161 |
| Sc-0628 | 38 | CIN1 | ASC-US | 33, 52 | Sc-0723 | 39 | CIN2 | - | 33, 52 | Observation | - | 224 |
| Sc-0717 | 28 | CIN2 | LSIL | 16, 34 | Sc-0748 | 28 | CIN2 | - | 16 | Observation | - | 70 |
| Sc-0632 | 26 | CIN2 | LSIL | 66 | Sc-0703 | 26 | CIN1 | - | 66 | Observation | - | 196 |
| Sc-0558 | 24 | CIN1 | LSIL | 42, 66 | Sc-0707 | 25 | CIN1 | - | 66, 68 | Observation | - | 413 |
| Sc-0597 | 31 | CIN2 | HSIL | 81 | Sc-0716 | 32 | CIN1 | - | 45, 58 | Observation | - | 329 |
| Sc-0415 | 48 | CIN1 | HSIL | 6 | Sc-0517 | 49 | CC | HSIL | 18, 81 | Observation | - | 308 |
| Sc-0611 | 29 | CC | ASC-US | 82 | Sc-0727 | 30 | - | NILM | negative | Observation | - | 273 |
| Sc-0664 | 33 | CIN3 | HSIL | 31, 58 | Sc-0737 | 34 | CIN3 | - | 31, 58 | Observation | - | 147 |
| *Foot note* : CC: chronic cervicitis; CIN: cervical intraepithelial neoplasia; NILM: negative for intraepithelial lesion or malignancy; ASC-US: atypical squamous cells of undermined significance; LSIL: low-grade squamous intraepithelial lesion; HSIL: high-grade intraepithelial lesion; ASC-H: atypical squamous cells, cannot exclude high-grade squamous intraepithelial lesion. | | | | | | | | | | | | |
