# Supplementary figures and images for: Changes to the cervicovaginal microbiota and cervical cytokine profile following surgery for cervical intraepithelial neoplasia
Source: Sci Rep. 2021 Jan 25;11:2156. doi: 10.1038/s41598-020-80176-6 (PMC7835242; doi:10.1038/s41598-020-80176-6)

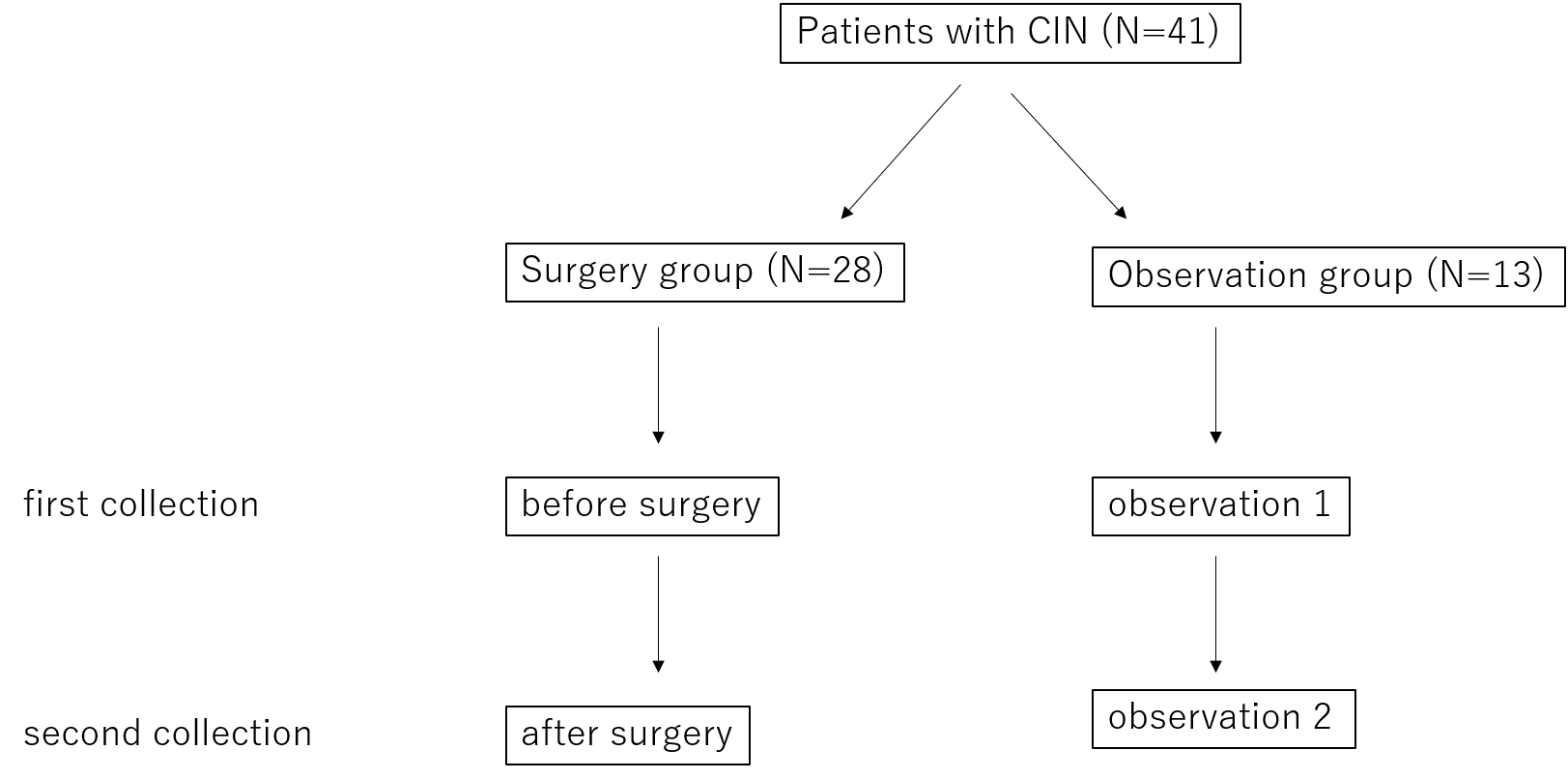

Supplement: Supplementary file 3 — Supplementary Figure 1. [file 41598_2020_80176_MOESM3_ESM.tif]

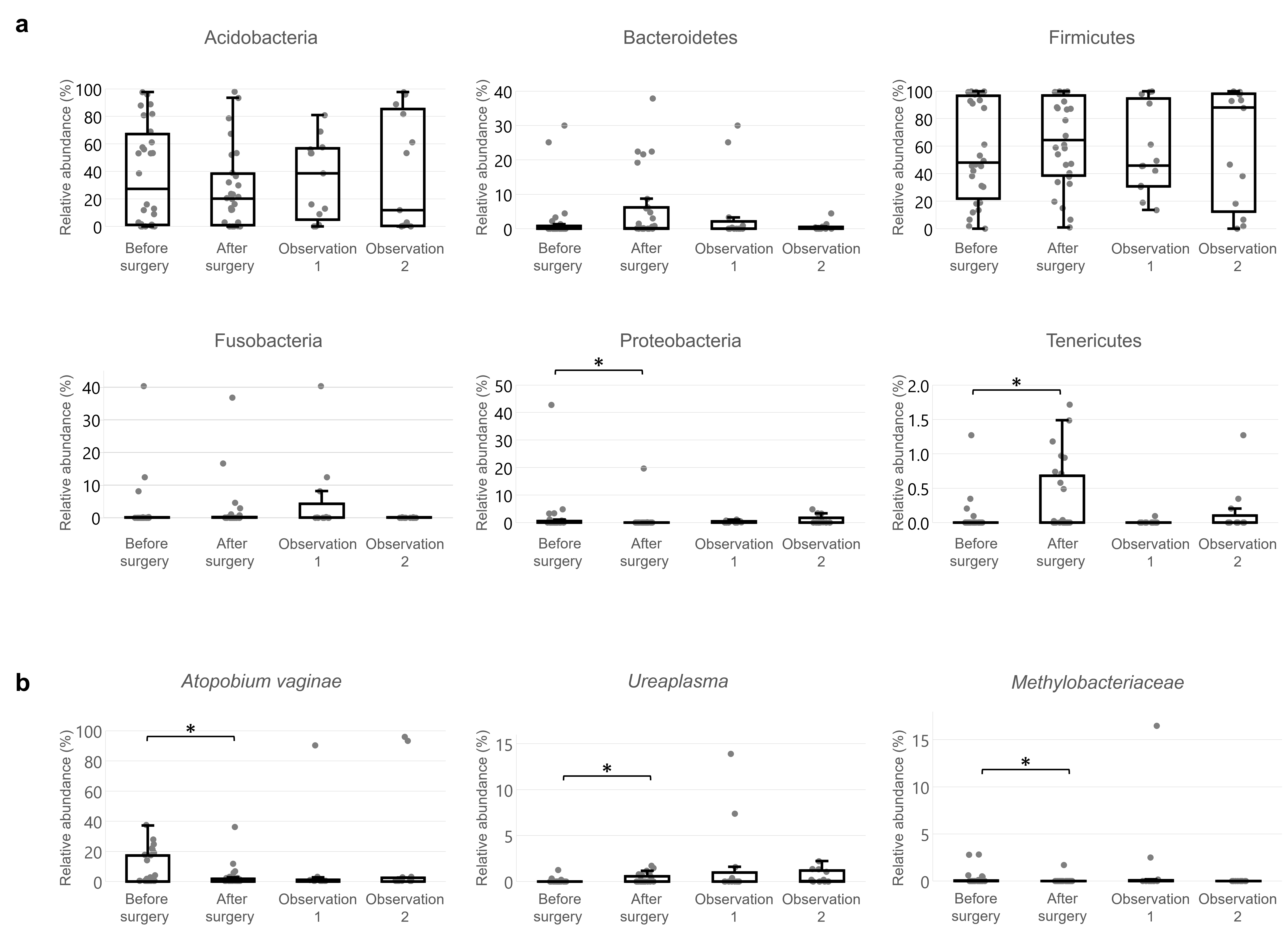

Supplement: Supplementary file 4 — Supplementary Figure 2. [file 41598_2020_80176_MOESM4_ESM.tif]
